# Mechanical Properties and Micro-Mechanisms of Chromite Sand Frozen Sand Molds Prepared by Ultrasonic Vibration Assistance

**DOI:** 10.3390/ma19122635

**Published:** 2026-06-18

**Authors:** Bailiang Zhuang, Haoqin Yang, Zhongde Shan, Zhuozhi Zhu, Di Ding

**Affiliations:** 1 Jiangsu Branch of China Academy of Machinery Science and Technology Group Co., Ltd., Changzhou 213000, China; zblalhl@outlook.com (B.Z.); zhuzzyc@163.com (Z.Z.); 13972511870@163.com (D.D.); 2 College of Materials Science and Technology, Nanjing University of Aeronautics and Astronautics, Nanjing 210016, China; 3 China Academy of Machinery Science and Technology Group, Beijing 100044, China; shanzd@cam.com.cn

**Keywords:** frozen sand molds, ultrasonic assisted, microstructure, freeze casting, sand performance, solidification mechanism

## Abstract

**Highlights:**

**Main findings**

- First, ultrasonic cavitation reduces water-rich and water-poor zones, enabling uniform moisture distribution through micro-liquid bridges.
- Second, ultrasonic vibration promotes fine and uniform ice crystals that form a continuous sand–ice network, effectively suppressing frost heave and microcracks.
- Third, ultrasonic-assisted preparation significantly boosts the tensile and compressive strengths of frozen sand preforms.

**Implications**

- First, introducing ultrasonic vibration-assisted technology into the mixing process of frozen sand preforms provides a new technical route for manufacturing high-quality frozen sand molds.
- Second, this research reveals the mechanisms of moisture migration and ice crystal growth under ultrasonic action, deepening the understanding of the solidification and forming laws of frozen sand preforms.
- Third, the research findings facilitate the industrialization of digital frozen sand green casting technology, reducing reliance on highly polluting resin sand in the aerospace and mechanical manufacturing sectors.

**Abstract:**

Frozen sand molds are the key material in digital frozen sand mold green casting technology, and their mechanical properties directly affect casting quality. Currently, these molds are primarily prepared by mechanical stirring, mixing, and compaction, which tend to cause imbalanced moisture adsorption and localized wet–dry differences, ultimately impairing the performance and quality of the castings. In this study, an ultrasonic vibration-assisted platform was established to prepare chromite sand frozen sand molds. By introducing ultrasonic vibration into the preparation process, a superior “sand grain–ice crystal” microstructure was constructed, leading to significantly enhanced mechanical properties. The tensile and compressive strengths were increased by approximately 10%, and the optimal process window for achieving the best mechanical performance of chromite sand was obtained.

## 1. Introduction

Sand mold casting is widely applied in aerospace, engineering machinery, and shipbuilding owing to its low cost and high adaptability, representing the dominant casting process [1,2,3]. Resin sand casting, the most prevalent technique, emits a lot of harmful fumes during pouring and generates non-recyclable waste sand [4,5]. Digital frozen sand mold green casting technology was proposed by the team of Shan Zhongde at Nanjing University of Aeronautics and Astronautics. After mixing with molding sand, the mixture is frozen below −25 °C into frozen sand preforms, which are then CNC-machined into high-precision molds. This technique features high precision, reusability, and environmental friendliness [6,7].

Frozen sand molds are molds created by freezing a mixture of sand and water. Frozen water serves as a binder, providing the necessary mold strength, and no harmful gases or odors are generated during the casting process [8,9]. After molten metal pouring, the ice crystal bonding bridges within the frozen sand mold are melted by the high-temperature molten metal, causing the mold to naturally collapse, which features reduced time and energy consumption. During the manufacturing process of frozen sand preforms, the uniform mixing of sand particles and water, along with the evolutionary mechanism of the water–ice phase interface in frozen sand molds, has a significant impact on the mechanical properties of the frozen sand preforms [10]. Zhang et al. [11] established thermo-hydro-mechanical coupling control equations for soft clay, analyzed the relationships among water migration, temperature distribution, and stress field variations during horizontal freezing, and revealed the driving effects of temperature potential energy and gravitational potential energy on water migration. Shi et al. [12] adopted the computational fluid dynamics (CFD) method to numerically simulate the migration behavior of water-based binders in frozen sand molds and systematically analyzed the influences of droplet size, injection frequency and ambient temperature on binder diffusion and migration. Tada et al. [13] studied the effects of grain size, morphology, and the substrate material of frozen sand molds on the mechanical strength of castings and found that introducing lead-free bismuth bronze into the frozen sand mold can effectively improve the thermal conductivity of the sand mold, while the good air permeability of the sand mold contributes to uniform solidification and grain refinement of the casting. Shan et al. [14] studied the variation laws of tensile strength and air permeability for frozen sand molds under different freezing temperatures and water contents and believed that the tensile strength and air permeability of frozen sand preforms gradually increase with the decrease in freezing temperature and the increase in water content. At present, frozen sand preforms are mainly prepared by mechanical stirring, but there are few studies on the sand mixing stage. It is difficult to control the uniformity of sand–water mixture preparation. The mechanical stirring preparation process easily leads to unbalanced water adsorption, resulting in local dry–wet differences, which directly affects the mechanical properties after freezing and ultimately affects the performance and quality of castings.

Ultrasonic vibration technology drives the workpiece to produce high-frequency micro-amplitude vibration through an ultrasonic transducer. In the material mixing process, this vibration can induce a cavitation effect inside the medium, effectively destroy particle agglomerates, and promote convective diffusion of various phases, thus significantly improving the uniformity of material distribution [15,16]. Younas et al. [17] investigated the influence of ultrasound-assisted ice-seeding on ice crystal formation. Through triggering ice nucleation, suppressing supercooling temperature, and controlling ice crystal size and shape, they found that this method, compared to conventional freezing, effectively achieves uniform ice crystal distribution and generates more regular pores in the structure. Kai et al. [18] reported that the cavitation effect of ultrasound can effectively break particle agglomerates in composite materials, improve particle dispersion, and significantly enhance microstructural uniformity, thereby improving the mechanical properties of ceramic particle-reinforced aluminum matrix composites when ultrasonic vibration is applied during their preparation. Han et al. [19] found that mechanical vibration, cavitation effect and acoustic streaming effect generated by ultrasonic vibration can improve the morphology of dendrite growth. Introducing ultrasonic vibration into the laser cladding process on the surface of 45 steel can effectively reduce micro-defects such as cracks, pores and interlayer separation in the workpiece. Feng et al. [20] conducted simulation analysis on ultrasonic cavitation parameters and found that when high-frequency ultrasonic vibration acts on a liquid medium, a large number of cavitation bubbles are continuously generated. These bubbles grow, oscillate, and collapse over ultrasonic cycles, thereby improving the uniformity of force distribution on the crushed workpiece. Huang et al. [21] analyzed the influence of ultrasonic vibration on the crystal plastic deformation of AISI 9310 steel and believed that ultrasonic vibration effectively promotes the interaction between dislocations and grain boundaries, optimizes grain boundary configuration, weakens deformation texture, reduces material flow stress, weakens strain hardening effect, and improves the plastic deformation ability and forming processing performance of materials.

At present, there are still few studies on ultrasonic vibration in particle systems with low water content and high solid-phase content, and its action mechanism has not formed a unified understanding. Especially in the preparation process of frozen sand preforms, the introduction effect and regulation mechanism of ultrasonic vibration lack systematic exploration and need further in-depth research. In summary, this paper built a test platform, applied ultrasonic vibration technology to the preparation process of frozen sand preforms, and studied the distribution characteristics of sand particles and water, as well as the evolution mechanism of the microstructure during the freezing process of sand preforms.

## 2. Experimental Equipment and Methods

### 2.1. Experimental Materials and Equipment

The sand particles of the frozen sand preform prepared in this paper are 70/140 mesh chromite sand produced by Tianjin Jinqin New Material Co., Ltd. (Tianjin, China). The main component of the sand particles is Cr_2_O_3_·Fe_2_O_3_, with a fine octahedral surface, mainly black massive or granular and semi-metallic luster.

**Figure 1:**
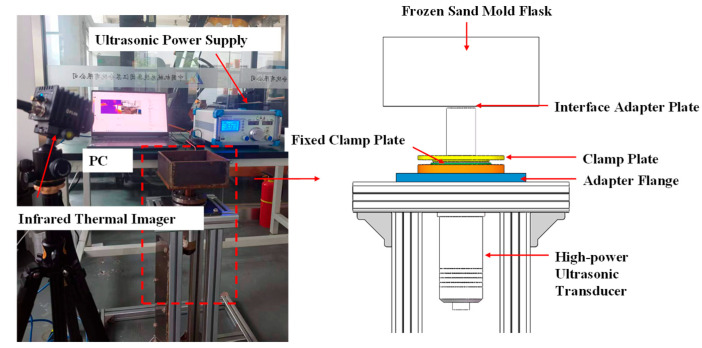
Experimental apparatus for ultrasonic-assisted preparation of frozen sand preforms.

The ultrasonic-assisted preparation apparatus for frozen sand preforms is shown in Figure 1, and it mainly consists of an ultrasonic transducer, vibration isolation plates, vibration absorption plates, a sand flask, an infrared thermal imager (Model A50, FLIR Systems, Inc., Wilsonville, OR, USA), and other components. The frozen sand flask has a rectangular cuboid structure with dimensions of 180 mm × 160 mm × 120 mm, and its total mass is 4400 g. During the preparation process, fine sand with a mesh size of 70/140 is mixed with deionized water in a mixer, and after thorough stirring, the mixture is added into the sand flask. An ultrasonic generator (Shenzhen Jidao Technology Co., Ltd., Shenzhen, China) was used to convert alternating current into high-power ultrasonic waves at a frequency of 20 kHz, which are then transmitted into the molding sand through a connecting flange to improve its uniformity and forming quality.

### 2.2. Experimental Scheme

**Figure 2:**
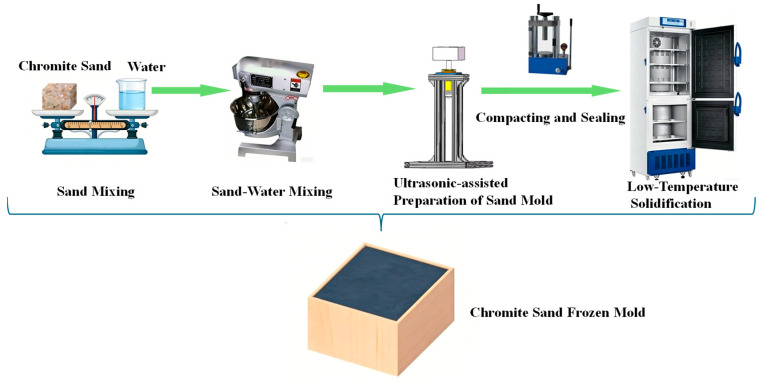
Process flow of ultrasonic-assisted preparation of frozen sand preforms.

The ultrasonic-assisted preparation process for frozen sand preforms is illustrated in Figure 2. The procedure is as follows:

(1) Weigh chromite sand and deionized water proportionally.
(2) Mechanically stir and mix the weighed chromite sand and water thoroughly for 20 min.
(3) Place the mixed sand into the sand flask and apply ultrasonic vibration technology to assist in preform preparation.
(4) Encapsulate and compact the mixture using a press.
(5) Place the compacted sand flask in an industrial freezer for low-temperature freezing treatment at −35 °C for 48 h.

This paper presents a comparative analysis of the performance between the frozen sand preforms and conventionally prepared frozen sand preforms.

To investigate the effects of water content, ultrasonic vibration time, and ultrasonic power on the mechanical properties of frozen sand preforms, the following experimental scheme was designed (Table 1).

**Figure 3:**
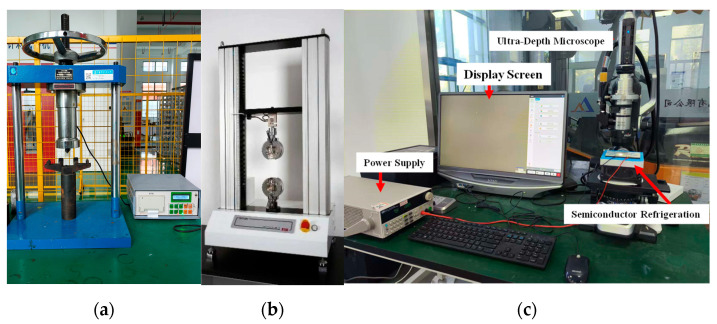
Experimental testing instrument. (**a**) Mechanical property testing platform for sand preforms. (**b**) XQY-II intelligent sand strength tester. (**c**) In situ characterization platform for low-temperature freezing.

A low-temperature freezing in situ characterization platform was established using an ultra-depth-of-field microscope (Model VHX-970FN, KEYENCE Co., Ltd., Shanghai, China), a thermal imager, and a semiconductor refrigeration system (Yileng Technology Co., Ltd., Shenzhen, China) to investigate the moisture distribution characteristics of frozen sand preforms and microstructure evolution during the freezing process. Conducting the macroscopic mechanical property tests of frozen sand molds [22], the tensile strength and compressive strength of frozen sand preforms were measured using a sand mold mechanical property testing platform and an XQY-II intelligent sand strength tester (Tairuida Instrument Technology Co., Ltd., Wuxi, China), as shown in Figure 3 below. This equipment requires calibration before use, and the calculation formulas for sand mold strength are pre-installed in the accompanying professional software. The semiconductor refrigeration system consists of an ultra-low-power semiconductor thermoelectric cooling chip, a fan heatsink, and a 12 V power supply, with an operating temperature range of –40 °C to +70 °C. Specific parameters are listed in Table 2.

## 3. Experimental Results and Analysis

### 3.1. Microscopic Distribution Characteristics of Ultrasonic-Assisted Prepared Frozen Sand Preforms

**Figure 4:**
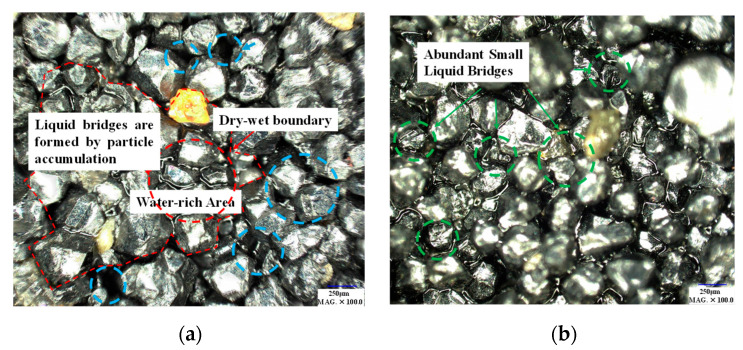
Microstructural distribution of 4.5 wt.% moisture chromite sand preforms at 100× magnification: (**a**) conventional preform preparation; (**b**) ultrasonic-assisted preform preparation.

According to the research on the green casting technology of frozen sand molds [23], chromite sand with a moisture content of 4 wt.%–5 wt.% achieves optimal air permeability and surface hardness simultaneously. Moderate air permeability facilitates the smooth escape of water vapor generated during molten metal pouring, effectively minimizing casting defects such as blowholes and misruns and yielding high-quality castings. Considering the practical production of frozen sand molds commonly adopts 4.5 wt.% moisture content, specimens with 4.5 wt.% moisture content are selected for microscopic characterization, and Figure 4 presents their microstructural distribution features. In conventionally prepared sand preforms, obvious “water-rich” and “water-poor” zones coexist, whereas the ultrasonically assisted frozen sand preforms exhibit a relatively uniform moisture distribution with finer liquid bridges. This comparison indicates that conventional preparation, which relies primarily on the shear force generated by stirring, can achieve large-scale material turning but fails to break micron-sized agglomerates. In contrast, ultrasonic vibration generates a cavitation effect: under the positive pressure of ultrasonic waves, cavitation bubbles rapidly compress, collapse, and eventually implode. This process effectively disrupts the fine agglomerates caused by water surface tension, improves the microscopic flow behavior of moisture, and directs the directional migration of water from enriched zones to depleted zones.

**Figure 5:**
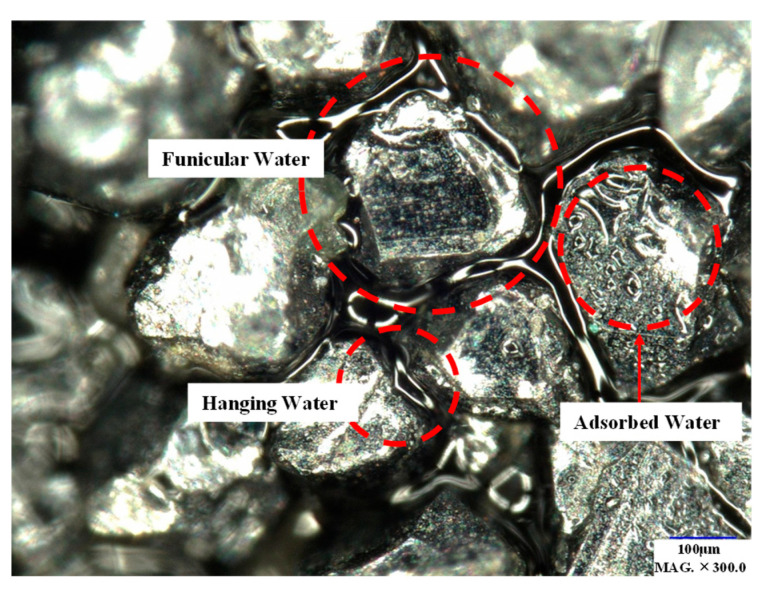
Main existing forms of water in 4.5 wt.% moisture chromite sand at 300× magnification.

Figure 5 illustrates the morphology of water in frozen sand preforms at 300× magnification, including pendular water formed independently by surface tension, funicular water resulting from the merging of multiple liquid bridges, and adsorbed water on the surface of the molding sand. The capillary force generated by these liquid bridges is the main cause of sand particle agglomeration and the formation of “wet agglomerates”. In frozen sand preforms, capillary force plays a key role in stabilizing the sand–water system prior to low-temperature freezing, and after freezing, its function is replaced by the bonding effect of ice crystal bridges. Observations reveal that the adsorbed water on the molding sand surface in ultrasonically assisted preforms exhibits a distinctly dispersed pattern due to vibration, forming abundant small droplets. This indicates that ultrasonic vibration effectively breaks the surface tension of the water system, allowing water to be more uniformly distributed within the interstices of the molding sand. The liquid bridges in these fine interstices provide ample capillary force, leading to tighter connections among sand particles.

**Figure 6:**
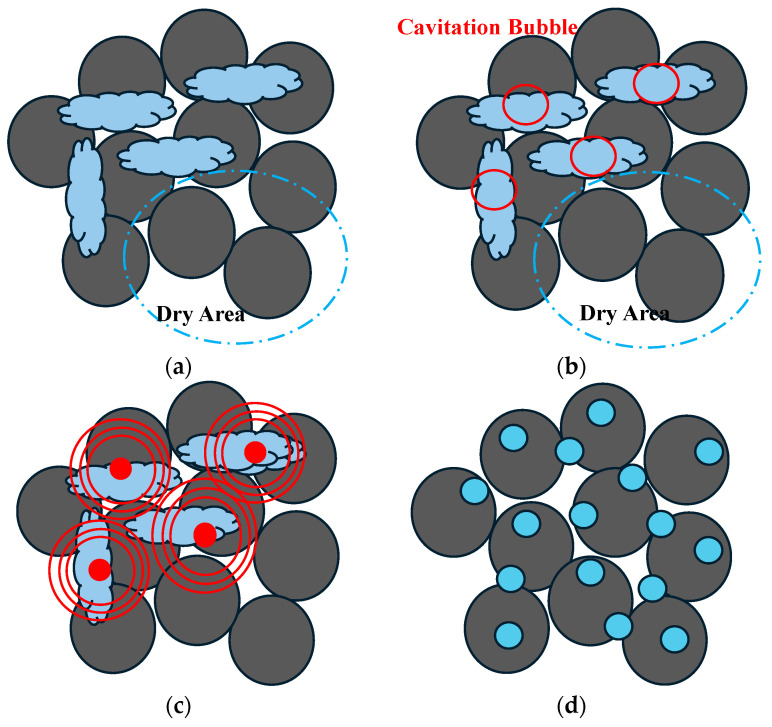
Schematic diagram of ultrasonic cavitation effect: (**a**) original wet sand agglomerate state; (**b**) cavitation bubble formation; (**c**) cavitation bubble collapse; (**d**) uniform dispersion of moisture.

In summary, the cavitation effect serves as the core driving force for sand particle dispersion and moisture migration during the ultrasonic-assisted preform preparation process. As illustrated in Figure 6, the instantaneous collapse of cavitation bubbles generates intense shock waves and micro-jets, which impose strong stresses on the sand particles that are sufficient to overcome capillary forces and break up fine sand agglomerates. Simultaneously, the high-pressure shock waves generated by cavitation disrupt the adsorption equilibrium between moisture and particle surfaces, causing the liquid bridges originally trapped at contact points to rupture and disperse into tiny water droplets. These droplets are then rapidly transported to the surfaces of dry sand particles under turbulent flow, forming new adsorbed water films.

**Figure 7:**
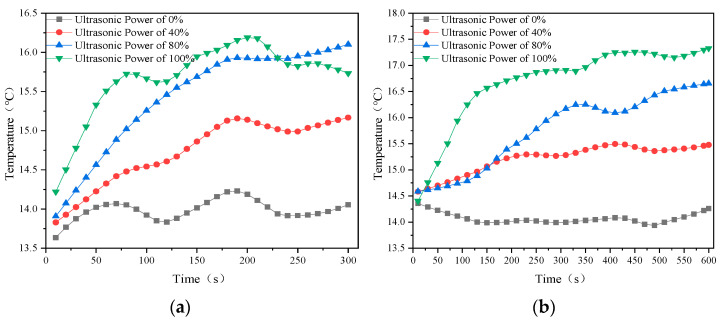

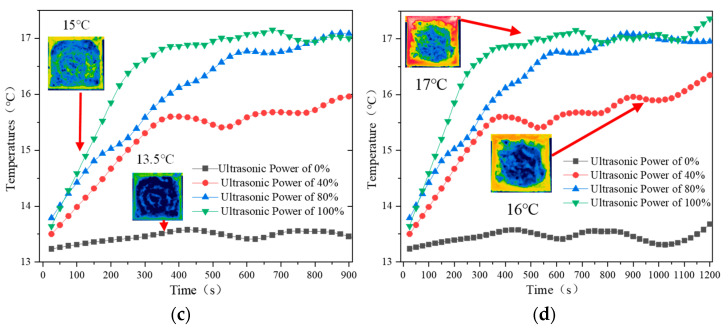
Temperature variation in chromite sand under different vibration times: (**a**) 5 min vibration; (**b**) 10 min vibration; (**c**) 15 min vibration; (**d**) 20 min vibration.

To further analyze the influence mechanism of ultrasonic vibration-assisted preform preparation on the uniformity of molding sand, an infrared thermal imager was used to observe the surface temperature variation under different ultrasonic vibration times and power levels. The results are presented in Figure 7 and summarized as follows.

(1) Higher ultrasonic power leads to a more significant rise in temperature of the sand preform. Regardless of the vibration time (5 min, 10 min, 15 min, or 20 min), the ultrasonic power is positively correlated with the preform temperature. The sand aggregate temperature is consistently the highest at 100% ultrasonic power. Under conventional preparation, the average internal temperature of the sand preform remains nearly unchanged, fluctuating around 13.5 °C. Higher ultrasonic power implies stronger energy input and generates more heat from cavitation and mechanical friction.
(2) The temperature rise rate is initially fast and then slows down, gradually approaching stability. As the vibration time increases from 5 min to 20 min, the temperature change in the sand preform exhibits a pattern of “rapid initial increase followed by a slower later increase”. For all power groups, the temperature climbs rapidly within the first 50 s, after which it enters a stage of fluctuating but slower increase. When the vibration time reaches 200 s, the temperature rise rate further decreases. After 15 min of vibration, the preform temperature becomes essentially stable, as the input ultrasonic energy roughly balances the heat dissipation rate, approaching a “dynamic equilibrium”. Further extending the vibration time has only a limited effect on the temperature increase.
(3) As the vibration time increases, the internal temperature distribution of the sand preform transitions from local non-uniformity to global uniformity. At 15 min, there exist a low-temperature zone at 13.5 °C and a high-temperature zone at 15 °C, with a temperature difference of approximately 1.5 °C. At 20 min, the temperature concentrates in the range of 16–17 °C, and the overall distribution is much more uniform. This indicates that the energy transfer effect generated by ultrasonic vibration tends to homogenize the internal temperature of the sand preform.

Overall, the temperature of the sand preform increases with higher ultrasonic power, rises rapidly and then stabilizes with prolonged vibration time, and the internal temperature distribution gradually becomes more uniform. Ultrasonic vibration is essentially a high-frequency mechanical vibration that is transmitted through the container wall to the entire fine sand system, generating multi-dimensional mechanical effects. This high-frequency vibration causes the “free” sand particles to migrate and also induces “elastic vibration” within the system, leading to periodic expansion and contraction of the interparticle voids. This creates a “pumping effect” that drives moisture migration toward dry regions, resulting in temperature variations around the preform and promoting internal temperature homogenization. As the vibration frequency increases, the high-frequency collisions and friction between particles raise the temperature, with the temperature rise being more pronounced closer to the inner wall of the sand flask. Meanwhile, the relative displacement and rearrangement of sand particles and liquid bridges under ultrasonic action facilitate denser particle packing and promote the penetration and spreading of tiny water droplets (dispersed by cavitation) between particles. This effectively overcomes the mixing limitation of fine sand with low water content, achieving better results than conventional preform preparation.

### 3.2. Microstructure Evolution Mechanism of Ultrasonic-Assisted Prepared Sand Preforms During Freezing

**Figure 8:**
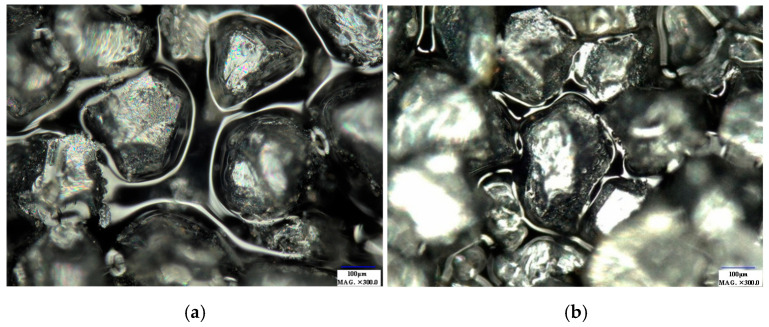

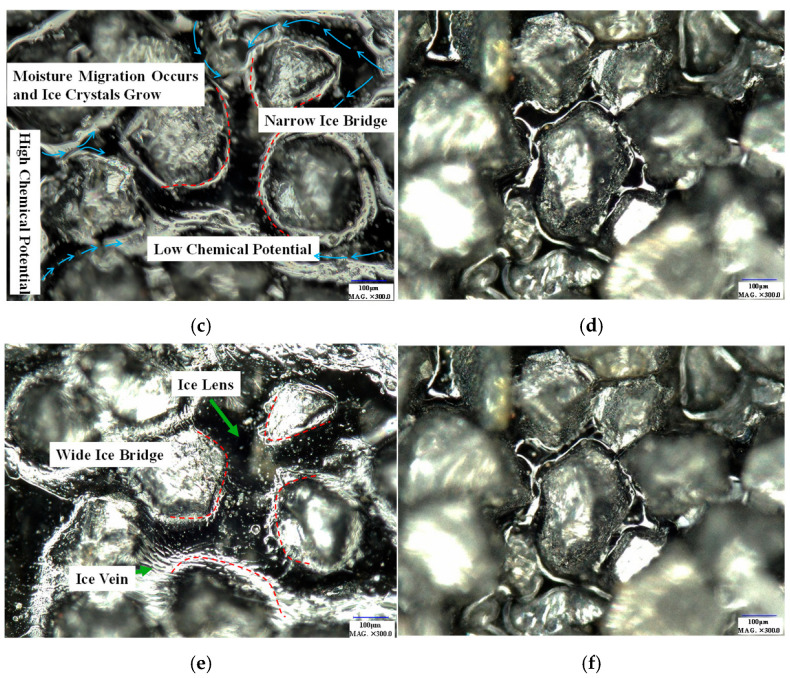
Comparison of phase transition in the low-temperature freezing region between conventionally and ultrasonically fabricated sand preforms with 4.5 wt.% moisture. (**a**) Initial water film morphology under conventional stirring. (**b**) Initial water film morphology under ultrasonic-assisted stirring. (**c**) Initial formation of ice lenses under conventional preparation. (**d**) Initial formation of ice lenses under ultrasonic-assisted preparation. (**e**) Full formation of ice lenses under conventional preparation. (**f**) Full formation of ice lenses under ultrasonic-assisted preparation.

The comparison of the phase transition process in the low-temperature freezing zone between conventionally prepared and ultrasonically assisted sand preforms is shown in Figure 7. Observations of the water film under normal conditions, as presented in Figure 8a,b, reveal that in conventionally prepared preforms, water is unevenly distributed within the fine sand, forming coarse and defect-laden liquid bridges. Although large ice crystals themselves may possess considerable strength, the interface between them and the sand particle skeleton is a weak link. The load transfer path is discontinuous, leading to severe stress concentration. In contrast, for the wet sand agglomerates prepared with ultrasonic vibration assistance, the funicular water is uniformly and dispersedly distributed in the form of extremely thin water films and tiny liquid bridges within the skeleton composed of 70/140 mesh fine sand, thereby forming an effective connecting network.

As the temperature gradually decreases, ice crystal bridges begin to form, accompanied by a noticeable moisture migration phenomenon. During the freezing process, when the temperature reaches −10 °C, “warm ice” forms in the ice layer of the sand preform. Under this condition, a rich liquid flow layer is generated. The ice crystals that form first have a lower chemical potential, which drives the unfrozen water still present in smaller pores nearby to migrate and accumulate toward the growing ice crystal front through the “capillary channels” provided by the liquid flow layer. This process continuously “feeds” a few ice crystals, enabling a secondary migration of moisture within the sand and ultimately forming macroscopically visible ice crystal bridges composed of pure ice. In the case of ultrasonic-assisted preform preparation (Figure 8d), the growth of each ice crystal is strictly confined by the surrounding sand particle skeleton, and the available amount of liquid water is very limited. As a result, the ice crystals cannot grow large, the water migration distance is extremely short, and no moisture accumulation occurs, effectively suppressing the secondary expansion of water-rich zones. In contrast, for conventionally prepared preforms (Figure 8c), the ice crystals that form first possess a lower chemical potential, driving unfrozen water in smaller pores to migrate and accumulate toward the growing ice crystal front through thin water films or capillary channels.

As the temperature decreases further, ice crystal bridges gradually form. In the case of ultrasonic-assisted preform preparation (Figure 8f), a large number of transparent ice crystals with small dimensions and uniform morphology appear inside, and almost no visible cracks or air pockets are observed. This confirms the occurrence of cavitation effects, as the ultrasonic vibration eliminates numerous cavitation bubbles and reduces ice crystal defects. These ice crystals fill the interparticle pores of the sand, acting like a “cementing agent” that firmly “welds” the originally loose sand particles together, forming a fine, interconnected ice crystal network. The entire wet sand agglomerate behaves as a composite material composed of sand particles and ice crystals. Because the ice crystals are small and uniform, the crystallization pressure generated during freezing is dispersed and balanced at the microscale, without forming concentrated stresses, thereby causing minimal disturbance to the sand particle skeleton.

In contrast, for conventionally prepared preforms (Figure 8e), ice crystals are large and irregular in shape, and numerous pores and defects can be observed within the ice crystals. Although large ice crystals themselves may not be weak, the interface between them and the sand particle skeleton is a vulnerable region. The load transfer path is discontinuous, leading to severe stress concentration.

**Figure 9:**
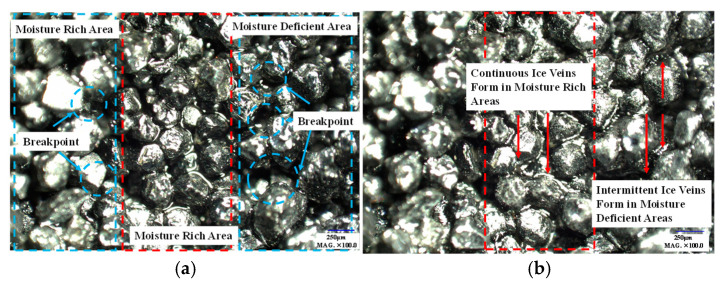
Water migration process in water-rich and water-poor zones during conventional preform preparation: (**a**) before freezing the sand preform; (**b**) after freezing the sand preform.

The macroscopic manifestation of the growth process of ice crystal bonding bridges in the sand preform is the change in the volume fractions of ice and water. To further analyze the phase transition behavior of moisture in water-rich and water-poor zones, it is observed that during conventional preform preparation, the boundary between dry and wet zones is clearly delineated, resulting in multiple “fractured” ice veins in the sand preform, as shown in Figure 9. These fractured ice veins are caused by the uneven distribution of free water inside the sand preform during material mixing and standing. Moisture locally accumulates to form water-rich zones, while other areas become water-deficient dry zones, leading to a significant dry–wet interface. Consequently, during freezing, the ice formation rates on both sides of the interface differ, and the combined effects of crystallization shrinkage and stress differences give rise to fractured ice veins. Such defects hinder the formation of a continuous liquid flow layer, thereby impeding the secondary migration of liquid bridges. Ultimately, after freezing, these discontinuous regions become the weakest points in terms of the mechanical properties of the sand preform.

**Figure 10:**
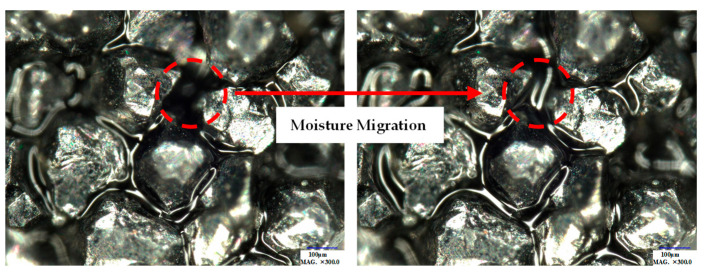
Water migration process during ultrasonic-assisted preform preparation.

Under ultrasonic action, as shown in Figure 10, as the freezing phase gradually forms, the pendular water and liquid bridges approach each other and undergo moisture migration. Meanwhile, it can be clearly observed that the liquid bridges within the sand preform migrate from relatively water-rich zones to relatively water-poor zones through the “capillary channels” formed by the liquid flow layer, completing the secondary migration process of liquid bridges. This further strengthens the liquid bridge network structure inside the entire sand preform, compensates for defective areas, and forms continuous liquid bridges, thereby helping to enhance the mechanical properties of the frozen sand preform.

In summary, ultrasonic-assisted preform preparation can effectively optimize the moisture distribution morphology, allowing water to be uniformly distributed as water films and micro-liquid bridges within the sand particle skeleton without obvious water-rich zones. During the freezing process, it enables grain refinement and global simultaneous nucleation, resulting in an ice crystal structure that is fine in size, uniformly distributed, and interconnected. The crystallization stress is evenly balanced, thereby suppressing frost heaves and reducing internal defects. In contrast, conventional preform preparation tends to cause uneven moisture distribution, with nucleation occurring preferentially in water-rich zones during freezing, accompanied by moisture migration that forms coarse ice lenses, which readily induce frost heaves and microcracks.

### 3.3. Mechanical Properties of Ultrasonic-Assisted Prepared Frozen Sand Preforms

**Figure 11:**
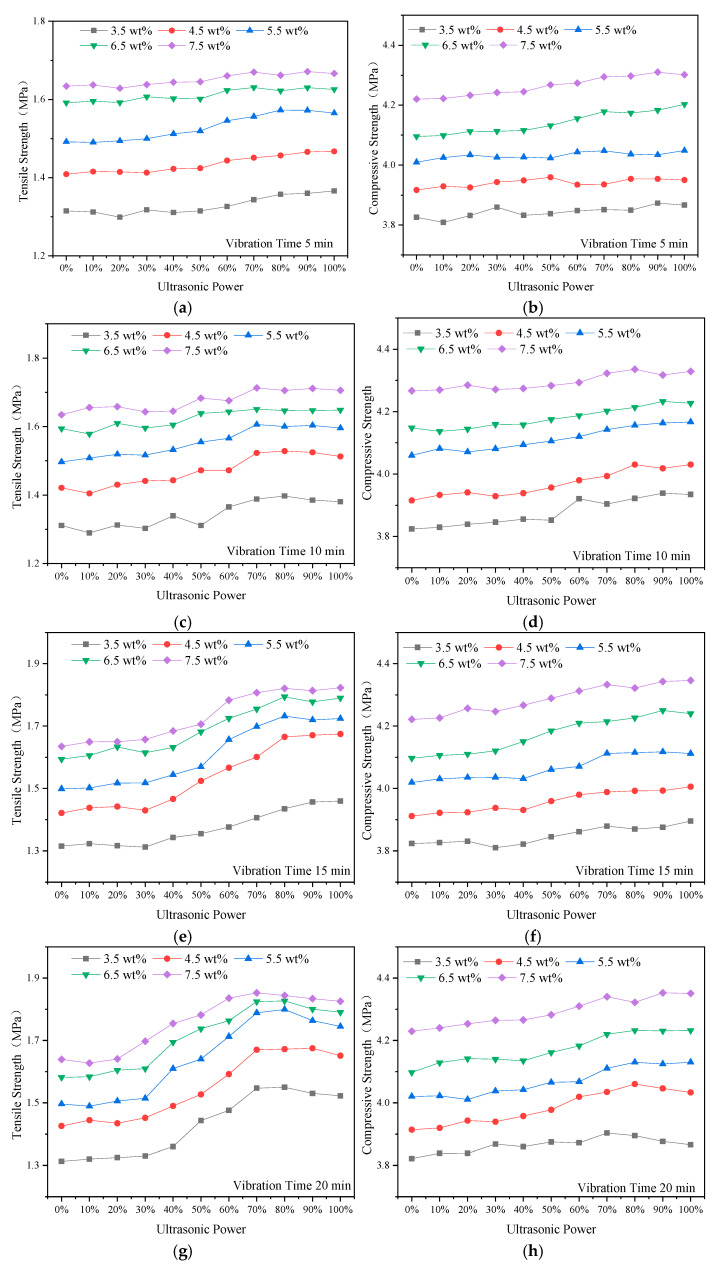

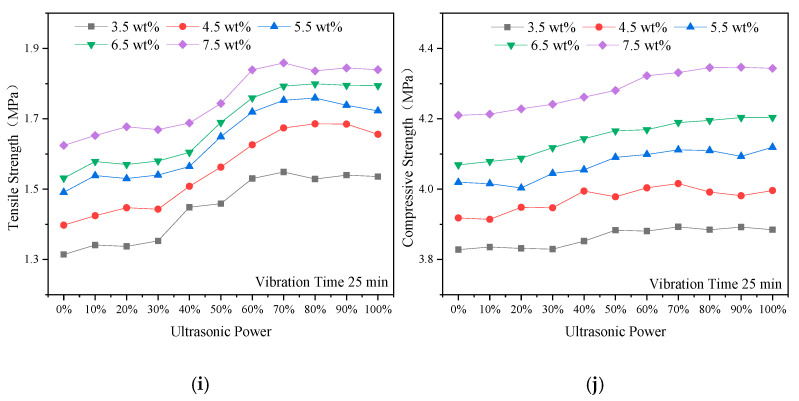
Variation in tensile and compressive strength of chromite sand under different conditions; (**a**) 5 min tensile strength; (**b**) 5 min compressive strength; (**c**) 10 min tensile strength; (**d**) 10 min compressive strength. (**e**) 15 min tensile strength; (**f**) 15 min compressive strength; (**g**) 20 min tensile strength; (**h**) 20 min compressive strength; (**i**) 25 min tensile strength; (**j**) 25 min compressive strength.

This paper compares the tensile strength and compressive strength of chromite sand under different water contents and different vibration times. The results are shown in Figure 11, and the observed patterns are as follows:

(1) Water content plays a dominant role in the mechanical properties of frozen sand preforms.

Regardless of changes in ultrasonic power and vibration time, the tensile and compressive strengths generally show an increasing trend with increasing water content. For example, at a vibration time of 15 min and an ultrasonic power of 0%, which can be regarded as a conventionally mixed frozen sand mold, when the water content of the frozen sand preform increases from 3.5 wt.% to 7.5 wt.%, the tensile strength rises from 1.35 MPa to 1.58 MPa. This result is close to the findings of Shi et al. [24], further validating the reliability of the present experimental results. When the ultrasonic power is 100% and the water content increases from 3.5 wt.% to 7.5 wt.%, the tensile strength increases from 1.47 MPa to 1.77 MPa. A comparison reveals that the increase in water content enhances tensile strength by about 20%, while ultrasonic vibration enhances tensile strength by about 10%. When the water content of the frozen sand preform increases from 3.5 wt.% to 7.5 wt.%, the compressive strength increases from 3.82 MPa to 4.22 MPa; at a water content of 5.5 wt.%, the compressive strength is 4.02 MPa. When the ultrasonic power is increased to 100%, the compressive strength rises from 3.89 MPa to 4.32 MPa. Thus, the mechanical properties are mainly influenced by the size and number of ice crystal bridges. As the water content inside the sand mold increases, the total number of ice crystal bridging bonds in the frozen sand preform increases correspondingly, leading to a significant increase in tensile/compressive strength. In contrast, ultrasonic vibration only affects the size and number of ice crystal bridges by modifying the moisture distribution, resulting in a limited increase in tensile/compressive strength.

(2) Effect of ultrasonic power.

Ultrasonic power exhibits a clear positive correlation with the tensile and compressive properties of frozen sand preforms. In the low-power range (0–30%), ultrasonic vibration provides very limited improvement in mechanical properties. In the medium-power range (30–70%), the mechanical properties enter a rapid growth phase, with tensile and compressive strengths increasing significantly as ultrasonic power rises. In the high-power range (70–100%), the growth of tensile and compressive strengths slows down and gradually stabilizes. The range of 70–80% (approximately 1500 W) represents the “turning point” for the performance enhancement of frozen sand preforms, reaching the optimal mechanical property zone. Further increases in ultrasonic power may lead to performance degradation due to excessive cavitation and bubble erosion.

(3) Effect of ultrasonic vibration time.

Under the same ultrasonic power and water content, a longer vibration time leads to higher mechanical properties. Both tensile and compressive strengths increase to varying degrees, and the rate of increase becomes more pronounced as the vibration time prolongs. When the vibration time reaches 20 min, the mechanical properties of the frozen sand preform essentially reach their maximum, indicating that at this point, the ultrasonic-assisted vibration has achieved a favorable mixing state inside the preform, and further increasing the vibration time no longer continuously enhances the tensile and compressive strengths.

**Figure 12:**
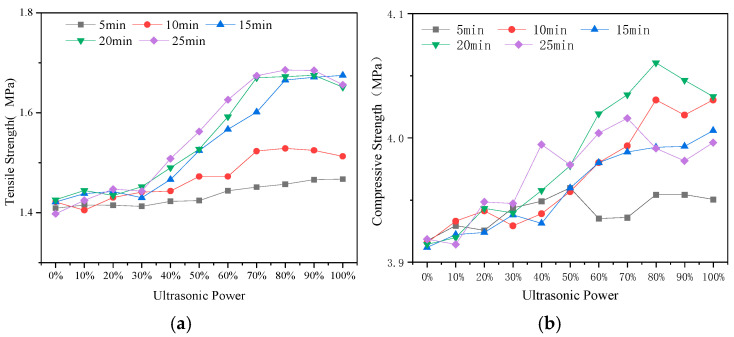
Variation curves of mechanical properties of chromite sand with 4.5 wt.% moisture content under different ultrasonic vibration durations: (**a**) tensile strength; (**b**) compressive strength.

Figure 12 shows the curves of tensile and compressive strength as a function of vibration time at a moisture content of 4.5 wt.%. Both tensile and compressive strengths are positively correlated with ultrasonic-assisted vibration power and vibration time, and the overall enhancement of tensile strength is superior to that of compressive strength. In the low-power range of 0–40%, the mechanical properties do not increase significantly with time. However, when the ultrasonic power exceeds the range of 40% to 70%, the curvature of the mechanical property increase becomes notably larger, with the most obvious improvement occurring in the interval of 15–20 min. When the ultrasonic power reaches 70–80%, the mechanical properties attain their maximum values. To further analyze the variation pattern of mechanical properties with temperature, it was found that the temperature change during the stirring process shows a positive correlation with the mechanical properties, consistent with the above trend. When the ultrasonic power reaches 70–90% at vibration times of 15 min and 20 min, both the mechanical properties and temperature reach their maximum values. At this point, the temperature curve shows that at ultrasonic powers of 80% and 100%, the temperature also reaches a maximum of 17 °C and does not change significantly with further increases in time. This consistency further demonstrates that ultrasonic-assisted vibration stirring is an effective approach for improving the performance of frozen sand molds.

## 4. Conclusions

(1) Ultrasonic-assisted preform preparation significantly improves the microscopic uniformity of chromite sand and water through the synergistic action of cavitation and high-frequency mechanical vibration. The cavitation effect disrupts water surface tension and separates sand–water agglomerates.
(2) High-frequency mechanical vibration further breaks up fine clusters and promotes relative motion among particles. This synergy effectively overcomes the mixing limitations inherent to fine sand with low water content, achieving superior uniformity compared to conventional preparation methods.
(3) Comparative observation of ice crystal bridge nucleation between conventionally and ultrasonically prepared preforms reveals distinct moisture distribution differences and clarifies the nucleation mechanism. During freezing, ultrasonic-assisted preparation exhibits substantially less moisture migration than conventional methods. Furthermore, the resulting ice bridges are notably finer and more uniformly distributed, avoiding the coarse, defect-laden ice lenses typical of conventional preforms.
(4) The proposed ultrasonic-assisted device effectively transmits high-frequency vibrational energy into the molding sand, improving the distribution of water and sand particles during preform preparation. Consequently, both the tensile and compressive strengths of the frozen sand preforms increase progressively with higher ultrasonic power and longer vibration time. The optimal mechanical properties for chromite sand preforms are achieved at an ultrasonic power of approximately 1500 W and a vibration time of 15 min.

## Figures and Tables

**Table 1:** Experimental scheme for ultrasonic-assisted preparation of chromite sand preforms.

	Water Content	Vibration Time	Ultrasonic Power Ratio
Parameters	3.5 wt.%, 4.5 wt.%, 5.5 wt.%, 6.5 wt.%, 7.5 wt.%	5 min, 10 min, 15 min, 20 min, 25 min	0%, 10%, 20%, 30%, 40%, 50%, 60%, 70%, 80%, 90%, 100%

**Table 2:** Specification of thermoelectric cooling chip.

Model	Rated Voltage (V)	Operating Current (A)	Cooling Power (W)	Temperature Difference (°C)	Size (mm)
TEC1-12705 (Yileng Technology Co., Ltd., Shenzhen, China)	12	5	45	45/60/40	40 × 40

## Data Availability

The original contributions presented in this study are included in this article. Further inquiries can be directed to the corresponding author.
